# The impact of the prolonged COVID-19 pandemic on the practice of psychosomatic medicine in Japan: a nationwide physician survey

**DOI:** 10.1186/s13030-025-00333-z

**Published:** 2025-07-09

**Authors:** Yukari Yamanaka, Kazuhiro Yoshiuchi, Nobuyuki Sudo, Chiharu Kubo, Shin Fukudo, Japanese Society of Psychosomatic Medicine, Ichiro Kusumi, Yuki Kako, Ken Sato, Motoyori Kanazawa, Shoichi Ebana, Keisuke Kawai, Takeaki Takeuchi, Mutsuhiro Nakao, Masahiro Hashizume, Shuichiro Maruoka, Hiroe Kikuchi, Hiroshi Kaneko, Chikara Yamaguchi, Yumiko Furui, Mikihiko Fukunaga, Atsuko Koyama, Makoto Hashizume, Hideaki Hasuo, Tetsuya Abe, Shuji Inada, Toshiyuki Tominaga, Hiroki Okada, Toshihide Harada, Kenji Kanbara, Akihiro Asakawa, Yoshio Kanemitsu, Sunao Matsubayashi

**Affiliations:** 1 Department of Stress Sciences and Psychosomatic Medicine, Graduate School of Medicine, The University of Tokyo, 7-3-1 Hongo, Bunkyo-Ku, Tokyo, Japan; 2 Department of Psychosomatic Medicine, Graduate School of Medical Sciences, Kyushu University, 3-1-1 Maidashi, Higashi-Ku, Fukuoka, Japan; 3 Nakamura Gakuen University, 5-7-1 Befu, Jonan-Ku, Fukuoka City, Fukuoka, Japan; 4 Graduate School of Medicine, Tohoku University, 2-1 Seiryo, Aoba, Sendai Japan

**Keywords:** COVID-19, Questionnaire survey, Psychosomatic medicine, Japan

## Abstract

**Background:**

The prolonged COVID-19 pandemic has significantly affected the clinical care and the mental health of patients in psychosomatic medicine. Between late 2021 and early 2022, the Japanese Society of Psychosomatic Medicine (JSPM) and the Japanese Society of Psychosomatic Internal Medicine (JSPIM) conducted a nationwide physician survey to assess these effects. The survey identified difficulties in outpatient and inpatient care, increased use of telemedicine, and rises in patient numbers and symptom severity. Due to inconsistent findings in prior studies on long-term mental health effects of the pandemic, a follow-up survey was needed.

**Methods:**

This study is the one-year follow-up survey conducted by JSPM and JSPIM. A cross-sectional, web-based survey was conducted among physicians of the two societies from December 21, 2022, to February 14, 2023. The questionnaire examined trends in outpatient and inpatient care, telemedicine use, and changes in the mental health of patients with psychosomatic disorders, eating disorders, adjustment disorders, mood disorders, and anxiety disorders. Descriptive statistical analyses were performed.

**Results:**

A total of 251 physicians responded. While outpatient numbers showed partial recovery, 28% of the respondents reported persistent declines compared to pre-pandemic levels. Telemedicine remained in use at 62% of their institutions, but 70% of the respondents reported difficulties in symptom assessment. Compared to the previous year, more respondents reported an increase in the number of patients across all surveyed disorders. Regarding the psychosocial factors that affected patients, fear of infection was the predominant factor for anxiety disorders, as in the previous survey, whereas restrictions on daily and social activities were the most influential for psychosomatic disorders, mood disorders, and adjustment disorders.

**Conclusions:**

The COVID-19 pandemic has had a lasting effect on patients receiving psychosomatic treatment. Outpatient numbers are gradually recovering, and telemedicine has contributed to the continuity of care. However, concerns about patient assessment in telemedicine persist. The impact of the prolonged pandemic on mental health appears to have evolved, with shifts in the psychosocial factors that influence different aspects of mental health deterioration. Future studies that incorporate clinical data will provide valuable insights into the long-term consequences of the pandemic and help guide future clinical practice.

**Supplementary Information:**

The online version contains supplementary material available at 10.1186/s13030-025-00333-z.

## Background

The Japanese Society of Psychosomatic Medicine (JSPM) and the Japanese Society of Psychosomatic Internal Medicine (JSPIM) conducted a nationwide survey of physicians from December 24, 2021, to January 31, 2022, to investigate changes in routine clinical practice and the impact of the COVID-19 pandemic on patients in psychosomatic medicine [1]. The study found that the global spread of the COVID-19 led to restrictions on routine outpatient and inpatient care and accelerated the adoption of telemedicine. Additionally, between 50 and 90% of physicians in psychosomatic medicine reported that the pandemic had affected patients with psychosomatic disorders, adjustment disorders, mood disorders, anxiety disorders, and eating disorders. These effects were associated with psychosocial factors such as fear of infection, social isolation due to restrictions on mobility, disruptions in school and family life, and reduced access to stress-coping strategies.

Even one year after the initial survey, the pandemic had not receded, with repeated cycles of infection surges and declines, leading to successive peaks in daily cases and death tolls. Until May 2023, when the legal classification of COVID-19 under the Infectious Diseases Control Law was revised, individuals who tested positive or were identified as close contacts of a person with COVID-19 were required to refrain from engaging in social and occupational activities for a designated period. Consequently, the pandemic has had long-term effects on the social lives and psychological well-being of the Japanese population.

Prospective longitudinal observational studies investigating the long-term effects of the COVID-19 pandemic on the general population and individuals with psychiatric disorders have yielded inconsistent findings. Some studies have reported that depressive and anxiety symptoms worsened during the early stages of lockdowns but subsequently improved as restrictions were lifted [2–5]. In contrast, another study suggested a progressive worsening of symptoms as lockdowns were repeatedly implemented [6]. Additionally, while slight improvements in mental health were observed over the two years following the onset of the pandemic, persistent issues such as heightened stress levels and reduced sleep duration remained [7]. Regarding the disorders examined in the present study, it has been reported that patients with adjustment disorders experienced a trajectory of anxiety and depressive symptoms similar to that of the general population. However, unlike the general population, their symptoms did not fully return to pre-lockdown levels even after the restrictions were lifted [5], suggesting that such patients may be more susceptible than others to the long-term effects of the pandemic.

Given these findings, we conducted the present study approximately one year after the initial survey to reassess the status of routine clinical practice in psychosomatic medicine and to investigate the long-term effects of the COVID-19 pandemic on patients through a nationwide survey of physicians in Japan.

## Methods

A nationwide questionnaire survey was conducted among physicians affiliated with JSPM and JSPIM. These societies were selected because they represent the primary professional organizations in Japan for physicians who treat patients requiring psychosomatic care.

The authors of this study identified key factors for evaluating the impact of the prolonged COVID-19 pandemic on routine clinical practice and on patients receiving psychosomatic medical care. They developed an original questionnaire based on these factors, drawing upon a similar study conducted approximately one year earlier [1]. An English translation of the questionnaire is provided in the Supplementary Table. The questionnaire consisted of five parts: (i) confirmation of consent; (ii) characteristics of the facility and department affiliation; (iii) influence of the prolonged COVID-19 pandemic on outpatient treatment; (iv) influence of the prolonged COVID-19 pandemic on inpatient treatment; and (v) influence of the prolonged COVID-19 pandemic on patients receiving psychosomatic medical care.

Questions regarding changes in routine treatment (iii) and (iv) primarily focused on the period during which the survey was conducted. Questions assessing the impact of the prolonged COVID-19 pandemic on patients (v) were answered based on the overall experiences of the respondents from 2022, approximately two years after the first wave of COVID-19 in Japan.

The survey was conducted online using Google Forms. Invitation emails were sent to all members of JSPM and JSPIM. At the time of the survey, 1,827 physicians belonged to JSPM and 985 to JSPIM. 585 physicians belonged to both societies. The response period was from December 21, 2022, to February 14, 2023. During this period, Japan experienced its eighth wave of the COVID-19 pandemic, with the highest number of daily deaths reporded compared to previous waves.

The institutional review boards of JSPM and JSPIM approved the study protocol, with consent implied by responding to the questionnaire. Descriptive statistics were used to summarize the responses.

## Results

### Respondent affiliations

A total of 251 physicians responded to the survey, with a response rate of 11.3%. The breakdown of their affiliated departments is as follows: 174 (69%) in psychosomatic medicine, 88 (35%) in internal medicine (excluding psychosomatic medicine), 69 (27%) in psychiatry, 19 in general medicine, 17 in pediatrics, 11 in palliative care, 9 in obstetrics and gynecology, and 16 in other specialties (including dermatology, rehabilitation, surgery, anesthesiology, otolaryngology, and psychosomatic dentistry). Some respondents reported affiliations with multiple departments. Regarding the size of the medical institutions where the respondents worked, 109 (43%) were from clinics, 52 (21%) were from general hospitals, 44 (18%) were from university hospitals, and 46 (18%) were from other types of hospitals.

### The influence of the prolonged COVID-19 pandemic on outpatient treatment

Among the respondents, 174 (69%) reported that the number of outpatients had returned to pre-pandemic levels. Seventy-one respondents (28%) reported a decrease compared to pre-pandemic levels, with approximately half reporting that the number remained below 80%.

**Fig.1:**
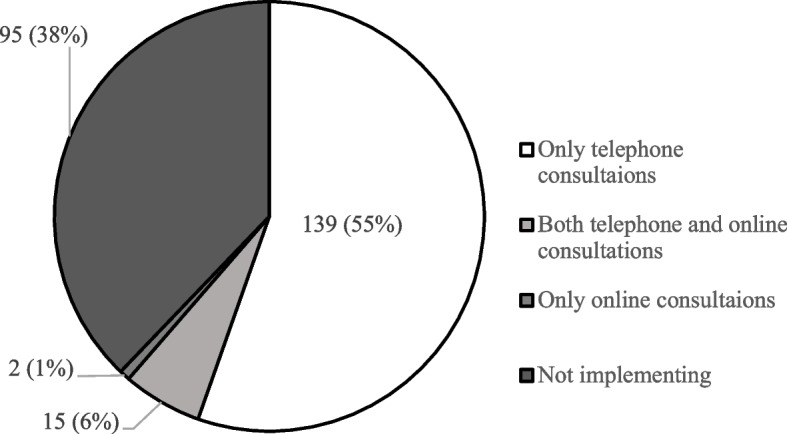
The status of telemedicine implementation

The status of telemedicine implementation is shown in Figure 1. The largest proportion of respondents (55%) conducted telephone consultations only, while 38% did not engage in any form of telemedicine. Tables 1 and 2 summarize the advantages and disadvantages of telemedicine. Aside from infection control, the most frequently cited advantage was the reduced rate of patient-initiated discontinuation of outpatient treatment. A total of 87% of the respondents recognized disadvantages associated with telemedicine, with 70% reporting difficulties in assessing symptoms and providing appropriate treatment. Nearly equal numbers of respondents reported challenges in the evaluation and intervention of both physical and mental symptoms.

**Table 1:** Responses about the advantages of telemedicine

	Number of respondents
Reduced rate of patient-initiated discontinuation of outpatient treatment	42
Improved patient satisfaction with treatment	29
Improved QOL	22
No advantages compared to face-to-face consultations	64
(Multiple responses allowed)

**Table 2:** Responses about the disadvantages of telemedicine

	Number of respondents
Difficulty in assessing physical symptoms and providing appropriate treatment	97
Difficulty in assessing mental symptoms and providing appropriate treatment	91
Difficulty in involving family members or other caregivers in treatment	32
Increased rate of patient-initiated discontinuation of outpatient treatment	6
No disadvantages compared to face-to-face consultations	20
(Multiple responses allowed)	

### The influence of the prolonged COVID-19 pandemic on inpatient treatment

Among the 99 respondents involved in inpatient care, 57 (58%) reported that the number of inpatients had returned to pre-pandemic levels, while 42 (42%) reported that it remained below pre-pandemic levels. Of these 42 respondents, nearly half indicated that their inpatient numbers were still below 80% of pre-pandemic levels.

### The influence of the prolonged COVID-19 pandemic on patients receiving psychosomatic medical care

Respondents providing care for patients with psychosomatic disorders, eating disorders, adjustment disorders, mood disorders, and anxiety disorders were asked about the impact of the prolonged COVID-19 pandemic on their patients and about associated psychosocial factors. Psychosomatic disorders refer to conditions among physical diseases in which psychosocial factors are closely involved in their onset and course and in which organic or functional impairment is observed. These disorders exclude somatic symptoms that are secondary to psychiatric disorders such as neurosis and depression.

**Fig. 2:**
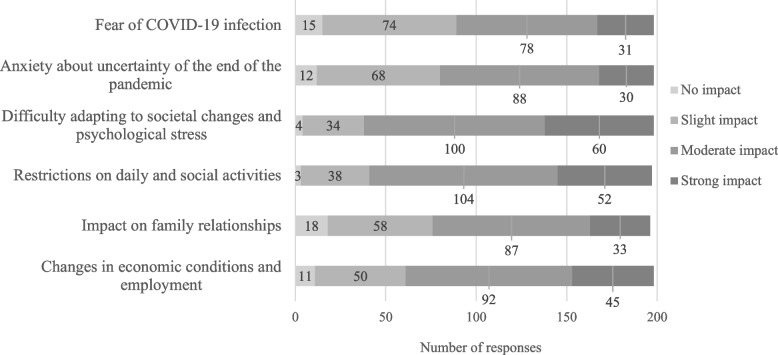
Responses about how the prolonged COVID-19 pandemic affected patients with psychosomatic disorders

Approximately 85% of the respondents reported a significant impact of the prolonged COVID-19 pandemic on patients with psychosomatic disorders. Approximately 65% reported an exacerbation of psychiatric symptoms, while about half noted a decline in the quality of life (QOL) of their patients and an increase in the number of patients. Difficulty adapting to societal changes, psychological stress, and lifestyle restrictions due to the COVID-19 pandemic were reported as major contributing factors (Figure 2). When asked specifically about physical symptoms and conditions linked to the prolonged pandemic, about 60% of the respondents reported cardiac and chest symptoms, nearly half reported headaches, a similar proportion noted abdominal symptoms, and approximately 40% observed obesity or weight gain.

**Fig. 3:**
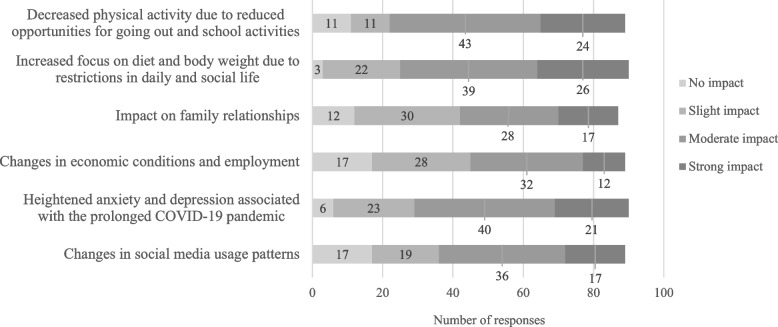
Responses about how the prolonged COVID-19 pandemic affected patients with eating disorders

Approximately 40% of respondents reported an increase in the number of patients with eating disorders, while a nearly equal proportion stated that they did not perceive a significant impact of the prolonged COVID-19 pandemic. Among those who acknowledged an impact of the prolonged pandemic on patients with eating disorders, about 65% reported an effect on binge eating and compensatory behaviors. The most frequently cited contributing factors were decreased physical activity due to movement restrictions and heightened preoccupation with body weight and food (Figure 3).

**Fig. 4:**
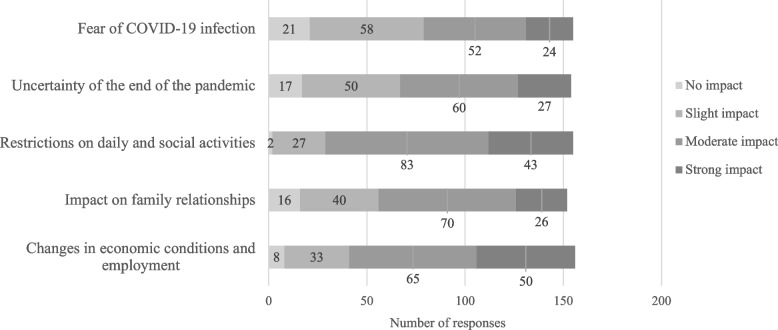
Responses about how the prolonged COVID-19 pandemic affected patients with adjustment disorders

**Fig. 5:**
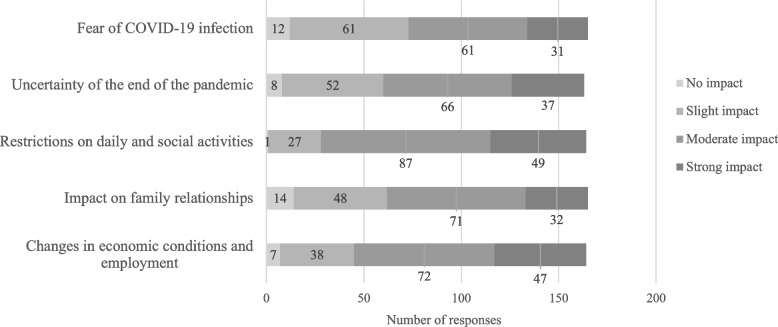
Responses about how the prolonged COVID-19 pandemic affected patients with mood disorders

**Fig. 6:**
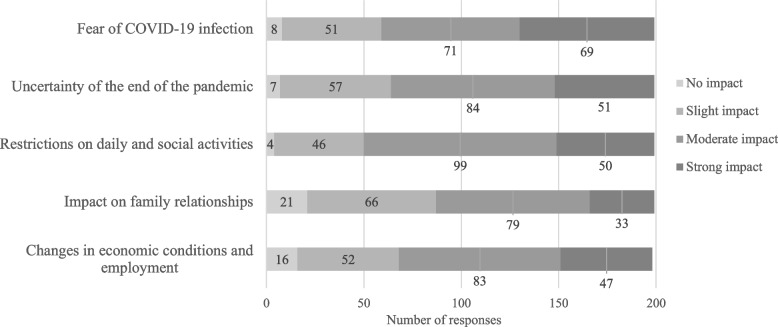
Responses about how the prolonged COVID-19 pandemic affected patients with anxiety disorders

The respondents reported an increase in the number of patients with an adjustment disorder, mood disorder, or anxiety disorder: 61%, 52%, and 62%, respectively, and a worsening of mental symptoms: 59%, 43%, and 53%, respectively. In addition, declines in QOL of patients with these disorders were noted: 59%, 46%, and 41%, respectively. Notably, approximately 90% of the respondents perceived an impact of the prolonged COVID-19 pandemic on anxiety disorders, the highest proportion among the five disorders examined. With respect to underlying psychosocial factors, respondents indicated that economic instability, employment concerns, and restrictions on daily life and social activities were major contributors to adjustment and mood disorders. In contrast, for anxiety disorders, approximately one-third of the respondents reported that fear of COVID-19 infection was a significant contributing factor (Figures 4, 5 and 6).

## Discussion

This study presents the findings of the second nationwide physician survey conducted by JSPM and JSPIM which investigated changes in clinical practice and the impact on patients in psychosomatic medicine during the COVID-19 pandemic.

In the initial survey conducted from December 24, 2021, to January 31, 2022, 46% of the respondents reported a decrease in the number of outpatients compared to the pre-pandemic period. In contrast, only 28% of respondents reported a decline in the current survey. A systematic review of mental health service use in Europe reported a decline in outpatient visits during the initial phase of the pandemic, and it was noted that among working-age adults, the number of outpatient visits increased during 2020 compared to the pre-pandemic period [8]. Although our study did not assess changes by age group, few institutions in Japan reported an increase in outpatient visits compared to the pre-pandemic level. Our findings suggest an increase in the number of outpatients in the year after our first survey. It appears that, similar to Europe, a decrease in outpatient visits occurred in Japan during the pandemic, but the recovery may have taken much longer.

The proportion of respondents using telemedicine was 66% in the previous survey and 62% in this survey, indicating that most medical institutions that introduced telemedicine have continued its use. While telemedicine has been considered beneficial in reducing infection risk and decreasing patient-initiated treatment discontinuation, approximately 70% of the respondents reported greater difficulty in assessing both physical and psychiatric symptoms and providing therapeutic interventions compared to in-person consultations. In the initial survey, 40% of the respondents expressed concerns about telemedicine, but this proportion increased significantly over the following year. Previous studies on telemedicine in psychiatric care have reported that remote and in-person consultations offer equally reliable diagnoses for conditions such as depression, ADHD, and schizophrenia [9]. However, other studies suggest that telemedicine is less suitable for establishing new therapeutic relationships [10]. As in-person consultations gradually resumed over the years following the pandemic, concerns about treating new patients appear to have emerged. Nevertheless, it is important to note that healthcare providers tend to prefer in-person consultations more strongly than patients do [9, 11]. Because the current survey targeted only physicians, concerns about telemedicine may have been overestimated. The primary reason COVID-19 affected clinical care appears to be the difficulty of maintaining routine medical services while implementing strict infection control measures during the pandemic. Similar challenges are likely to arise in the event of a future pandemic. Notably, our study revealed that the COVID-19 pandemic prompted the broader adoption of telemedicine. Although this study also highlighted concerns among physicians regarding telemedicine, its use along with conventional modalities, even if only on a temporary basis, may be effective for mitigating the decline in outpatient visits observed in the early phase of the pandemic.

Regarding the impact of the prolonged pandemic on patients, a comparison of the previous and current surveys revealed an increase in the proportion of respondents reporting more cases of the disorders included in the study: from 37 to 49% for psychosomatic disorders, 18% to 37% for eating disorders, 45% to 61% for adjustment disorders, 47% to 52% for mood disorders, and 53% to 62% for anxiety disorders. While the previous survey asked all respondents about all disorders, the current survey only included those actively involved in treating each disorder to enable a more accurate assessment. This methodological difference may have led to an overestimation of the increase in patient numbers, particularly for eating disorders, as treatment facilities specializing in these disorders are limited. However, 83–92% of the respondents in this survey were engaged in treatment for the other four disorders, suggesting that the risk of overestimation was relatively small.

To further investigate the perceived impact of the pandemic on patients, an additional survey was conducted beyond assessing the increase in the number of patients. For the disorders other than eating disorders, approximately 40–60% of the respondents reported a decline in QOL. A systematic review of studies conducted in Europe that examined changes in mental health among the general population and individuals with psychiatric disorders during the pandemic found no consistent trends in general psychopathology, depressive and anxiety symptoms, or eating disorder symptoms before and after the pandemic. However, a significant worsening of social functioning in psychiatric patients during the post-pandemic period has been reported with moderate certainty [8], which partially aligns with the findings of this study.

Regarding the psychosocial factors underlying the impact of the prolonged COVID-19 pandemic on patients, the initial survey suggested that fear of infection was the most influential factor, followed by social isolation due to movement restrictions. The current survey, which assessed these factors separately for each disorder, found that fear of COVID-19 infection remained the most significant factor influencing anxiety disorders, consistent with the previous survey. In contrast, for psychosomatic disorders, adjustment disorders, and mood disorders, chronic stress related to pandemic restrictions on daily and social life, economic instability, and difficulties in adapting to societal changes emerged as the predominant factors. A survey of university students in Japan indicated that initial concerns focused on financial issues and infection control measures. However, as the pandemic continued, psychological stress and dissatisfaction with daily life restrictions became more prominent [12]. Similarly, a study conducted across 11 European countries reported that in the early phase of the pandemic, a history of a psychiatric disorder was the primary risk factor for adjustment disorder symptoms, whereas after one and a half years, economic factors such as income reduction had a greater impact [13]. These longitudinal studies suggest that the factors that influenced mental health during the COVID-19 pandemic shifted over time, and our two surveys support this notion. Research has shown that social contact has a stronger positive impact on mental health when conducted in person rather than digitally [14, 15]. Additionally, increased face-to-face interactions have been linked to improvements in adjustment disorder symptoms during the pandemic, whereas digital communication has not shown the same effect [13]. Although in-person communication has increased since the early post-pandemic period, it has not yet returned to pre-pandemic levels, potentially exerting a long-term impact on mental health. A study comparing Japanese and Polish university students found that Japanese students exhibited greater fear of COVID-19 than their Polish counterparts and that this fear was negatively correlated with interdependent happiness [16]. While the specific items in the Fear of COVID-19 Scale that contributed to higher scores among Japanese participants were not identified, the continued impact of COVID-19-related fear on patients with anxiety disorders nearly three years after the first domestic case in Japan suggests that cultural factors may have played a role.

This study has several limitations. First, the response rate was low, 251 respondents, making it difficult to generalize the findings to all clinicians involved in psychosomatic medicine in Japan. Second, we allowed respondents to select multiple specialties in the questionnaire, which made it difficult to examine how the responses varied by specialty, such as psychosomatic medicine, other internal medicine specialties, or psychiatry. Moreover, we did not collect background information on respondents other than their specialty and the type of institution in which they worked. Physicians’ perspectives on telemedicine may vary depending on factors such as years of clinical experience or age, which may have limited the scope of our interpretations. These limitations indicate potential areas for improvement in how background information was collected. Third, this study relied on a survey of the experiences and perceptions of clinicians rather than objective clinical data such as medical records. Consequently, even quantitative findings, such as changes in patient numbers, may not be entirely reliable. Finally, although this study was a follow-up done one year after the previous survey, it was not originally designed as a longitudinal study. While both surveys targeted members of JSPM and JSPIM and the distribution of medical institutions remained similar, the degree of overlap in respondents between the two surveys is unclear, raising concerns about the validity of direct comparisons.

## Conclusions

The COVID-19 pandemic has had a significant impact on routine clinical practice; however, the number of outpatients and inpatients appears to have returned to pre-pandemic levels. In medical institutions that have adopted telemedicine, years of experience have clarified both its advantages and limitations, highlighting concerns about assessment and therapeutic intervention. Additionally, the prolonged pandemic has likely contributed to an increase in the number of patients, worsening psychiatric symptoms, and a decline in QOL. The psychosocial factors that influenced these effects may have changed over the past year, particularly in patients with psychosomatic disorders, mood disorders, and adjustment disorders. Conducting patient-based studies or analyzing medical records for each disorder will provide a more precise understanding of the long-term impact of the pandemic, offering valuable insights for clinical practice.

## Supplementary Information

Supplementary Material 1. Supplementary table. English translation of the questionnaire originally developed for this study.

## Data Availability

We cannot share our data because we did not obtain the consent of the respondents to share their data.
