# Supplementary material for: The impact of the prolonged COVID-19 pandemic on the practice of psychosomatic medicine in Japan: a nationwide physician survey
Source: Biopsychosoc Med. 2025 Jul 9;19:11. doi: 10.1186/s13030-025-00333-z (PMC12239389; doi:10.1186/s13030-025-00333-z)
Supplement: Supplementary file 1 — Supplementary Material 1. Supplementary table. English translation of the questionnaire originally developed for this study. [file 13030_2025_333_MOESM1_ESM.docx]

Research on the impact of the COVID-19 pandemic on psychosomatic medicine (especially stress-related disorders)

[English translation]

(i) Confirmation of consent

Are you a physician affiliated with the Japanese Society of Psychosomatic Medicine or the Japanese Society of Psychosomatic Internal Medicine, and currently engaged in clinical practice?

- Yes
- No

Do you understand the outline of this study and agree to participate, understanding that it is impossible to withdraw your consent after submission?

- I agree to participate in this research.
- I do not agree to participate in this research.

(ii) Characteristics of the facility and department affiliation

1. Please select all societies to which you belong.

- the Japanese Society of Psychosomatic Medicine
- the Japanese Society of Psychosomatic Internal Medicine

1. Please select all departments to which you belong.

- Psychosomatic medicine
- Internal medicine other than psychosomatic medicine
- Psychiatry
- Obstetrics and gynecology
- Pediatrics
- Dermatology
- General medicine
- Palliative care
- Other（please specify）

1. What type of medical facility do you belong to?

- University hospital
- General hospital
- Hospital other than university or general hospital
- Clinic

(iii) Outpatient care during the prolonged COVID-19 pandemic

1. Has the number of outpatients in your department returned to pre-pandemic levels?

- Yes
- No

1. If you answered "No" to the previous question, what is the approximate percentage of your current outpatient volume compared to pre-pandemic levels?

（Enter a number as a percentage）%

1. Are you implementing telemedicine (telephone or online consultations)?

- Both telephone and online consultations
- Only telephone consultations
- Only online consultations
- Not implementing (Skip to Question 9)

1. Besides reducing infection risk, what advantages of telemedicine do you perceive?
   - No advantages compared to face-to-face consultations
   - Reduced rate of patient-initiated discontinuation of outpatient treatment
   - Improved quality of life (QOL) in patients
   - Improved patient satisfaction with treatment
   - Other (please specify)
2. What disadvantages of telemedicine do you perceive?
   - No disadvantages compared to face-to-face consultations
   - Increased rate of patient-initiated discontinuation of outpatient treatment
   - Difficulty in assessing physical symptoms and provide appropriate treatment
   - Difficulty in assessing mental symptoms and provide appropriate treatment
   - Difficulty in involving family members or other caregivers in treatment
   - Other (please specify)

(iv) Inpatient care during the prolonged COVID-19 pandemic

1. Are you engaged in inpatient care? (Please answer not about COVID-19 specific hospitalizations, but routine inpatient care in your department)?
   - Yes
   - No (Skip to Question 12)
2. Has the number of inpatients in your department returned to pre-COVID-19 levels?
   - Yes
   - No
3. If you answered "No" to the previous question, what is the approximate percentage of your current inpatient volume compared to pre-COVID-19 levels?

（Enter a number as a percentage）%

(v) Influence of the prolonged COVID-19 pandemic on patients receiving psychosomatic medical care

1. Do you treat the following disorders in your routine clinical practice?
   - Psychosomatic Disorders
   - Eating Disorders
   - Adjustment Disorders
   - Mood Disorders
   - Anxiety Disorders
2. Please answer Questions 13-16 only if you are engaged in the treatment of psychosomatic disorders. If not, please skip to Question 17.

How has the prolonged COVID-19 pandemic affected patients with psychosomatic disorders?

- - The number of patients with psychosomatic disorders has increased.
  - The patients’ physical symptoms and test findings have worsened.
  - The required dosage of medication has increased for the management of psychosomatic disorders.
  - The patient’s mental state has been affected, leading to the onset or exacerbation of depressive and anxiety symptoms.
  - The patient’s quality of life (QOL) has declined.
  - No significant impact (Skip to Question 17)

1. To what extent do you recognize the following psychological factors have influenced patients with psychosomatic disorders by the prolonged COVID-19 pandemic?
   - Fear of COVID-19 infection
   - Anxiety about uncertainty of the end of the pandemic
   - Difficulty adapting to societal changes and psychological stress caused by the pandemic

(Each factor rated as: No impact, Slight impact, Moderate impact, Strong impact)

1. To what extent do you recognize the following social factors have influenced patients with psychosomatic disorders by the prolonged COVID-19 pandemic?
   - Restrictions on daily and social activities
   - Impact on family relationships
   - Changes in economic conditions and employment

(Each factor rated as: No impact, Slight impact, Moderate impact, Strong impact)

1. What physical symptoms or conditions do you recognize have been affected by the prolonged COVID-19 pandemic?
   - Abdominal symptoms (e.g., abdominal pain, bloating, constipation, diarrhea)
   - Cardiac and chest symptoms (e.g., palpitations, chest pain)
   - Primary headaches (e.g., tension-type headache, migraine)
   - Diabetes
   - Hypertension
   - Dyslipidemia
   - Obesity and weight gain
   - Respiratory diseases (e.g., asthma)
   - Orthostatic dysregulation
   - Atopic dermatitis
   - Menopausal disorders
   - Premenstrual syndrome (PMS) / Premenstrual dysphoric disorder (PMDD)
   - Postpartum or perinatal depression
   - Other (please specify)
2. Please answer Questions 17-18 only if you are engaged in the treatment of eating disorders. If not, please skip to Question 19.
   How has the prolonged COVID-19 pandemic affected patients eating disorders?
   - The number of patients with eating disorders has increased.
   - The number of patients requiring hospitalization due to severe anorexia nervosa has increased.
   - Weight loss in patients with anorexia nervosa has worsened.
   - Binge eating and compensatory behaviors have worsened.
   - No significant impact (Skip to Question 19)
3. To what extent do you recognize the following psychological and social factors have influenced patients with eating disorders due to the prolonged COVID-19 pandemic?
   - Decreased physical activity due to reduced opportunities for going out and extracurricular activities
   - Increased focus on diet and body weight due to restrictions in daily and social life
   - Impact on family relationships
   - Changes in economic conditions and employment
   - Heightened anxiety and depressive mood associated with the prolonged COVID-19 pandemic
   - Changes in social media usage patterns

(Each factor rated as: No impact, Slight impact, Moderate impact, Strong impact)

1. Please answer Questions 19-20 only if you are engaged in the treatment of adjustment disorders. If not, please skip to Question 21.
   How has the prolonged COVID-19 pandemic affected patients with adjustment disorders?
   - The number of patients with adjustment disorders has increased.
   - The patients’ mental symptoms have worsened.
   - The patient’s QOL has declined.
   - No significant impact (Skip to Question 21)
2. To what extent do you recognize the following psychological and social factors have influenced patients with adjustment disorders due to the prolonged COVID-19 pandemic?
   - Fear of COVID-19 infection
   - Uncertainty of the end of the pandemic
   - Restrictions on daily and social activities
   - Impact on family relationships
   - Changes in economic conditions and employment

(Each factor rated as: No impact, Slight impact, Moderate impact, Strong impact)

1. Please answer Questions 21-22 only if you are engaged in the treatment of mood disorders. If not, please skip to Question 23.
   How has the prolonged COVID-19 pandemic affected patients with mood disorders?
   - The number of patients with mood disorders has increased.
   - The patients’ mental symptoms have worsened.
   - The patient’s QOL has declined.
   - No significant impact (Skip to Question 23)
2. To what extent do you recognize the following psychological and social factors have influenced patients with mood disorders due to the prolonged COVID-19 pandemic?
   - Fear of COVID-19 infection
   - Uncertainty of the end of the pandemic
   - Restrictions on daily and social activities
   - Impact on family relationships
   - Changes in economic conditions and employment

(Each factor rated as: No impact, Slight impact, Moderate impact, Strong impact)

1. Please answer Questions 23-24 only if you are engaged in the treatment of anxiety disorders. If not, please skip to Question 25.
   How has the prolonged COVID-19 pandemic affected patients with anxiety disorders?
   - The number of patients with anxiety disorders has increased.
   - The patients’ mental symptoms have worsened.
   - The patient’s QOL has declined.
   - No significant impact (Skip to Question 25)
2. To what extent do you recognize the following psychological and social factors have influenced patients with anxiety disorders due to the prolonged COVID-19 pandemic?
   - Fear of COVID-19 infection
   - Uncertainty of the end of the pandemic
   - Restrictions on daily and social activities
   - Impact on family relationships
   - Changes in economic conditions and employment

(Each factor rated as: No impact, Slight impact, Moderate impact, Strong impact)

1. How has the prolonged COVID-19 pandemic affected your clinical practice? Please share any observations you may have.
2. What impacts have you observed in your patients due to the prolonged COVID-19 pandemic?
